# Evaluation of Thymic Output and Regulatory T Cells in Kidney Transplant Recipients with Chronic Antibody-Mediated Rejection

**DOI:** 10.1155/2021/6627909

**Published:** 2021-02-09

**Authors:** Abbas Shahi, Saeedeh Salehi, Shima Afzali, Ladan Gol Mohammad Pour Afrakoti, Marzie Esmaeili, Farzaneh Bagherpour, Ziba Aghsaeifard, Sanaz Dehghani, Gholamreza Pourmand, Ali Akbar Amirzargar

**Affiliations:** ^1^Department of Immunology, School of Medicine, Tehran University of Medical Sciences, Tehran, Iran; ^2^Student's Scientific Research Center, Tehran University of Medical Sciences, Tehran, Iran; ^3^Organ Procurement Unit, Sina Hospital, Tehran University of Medical Sciences, Tehran, Iran; ^4^Department of Internal Medicine, School of Medicine, Sina Hospital, Tehran University of Medical Sciences, Tehran, Iran; ^5^Department of Urology, School of Medicine, Sina Hospital, Tehran University of Medical Sciences, Tehran, Iran

## Abstract

**Background:**

Regulatory T cells (Tregs) and recent thymic emigrants (RTEs) have an essential role in the regulation of allogeneic immune responses. However, their mechanisms of action in chronic antibody-mediated rejection (cAMR) are still unclear. In this study, we aimed to compare Treg and RTE levels between stable graft function (SGF) patients and cAMR subjects after kidney transplantation.

**Method:**

Mononuclear cells (MNs) were separated from peripheral blood, and flow cytometry analysis was performed for detection of CD4^+^ and CD25^high^ as Treg markers and CD4^+^, CD31^+^, and CD45RA^+^ as RTE immunophenotyping markers.

**Result:**

The level of peripheral Treg cells was significantly lower in cAMR subjects in comparison to stable graft function patients. Moreover, SGF patients who had received cyclosporine A had a higher level of Treg in comparison to the tacrolimus recipients. Nevertheless, the RTE level between SGF and cAMR patients did not show any significant differences.

**Conclusion:**

It seems that Treg cells are significantly associated with transplant outcomes in cAMR patients, and prescribed immunosuppressive drugs can influence the frequency of this crucial subset of T cells. Although these drugs are beneficial and inevitable for allograft maintenance, more investigations are needed to elucidate their complete effects on different immune cell subsets which some of them like Tregs are in favor of transplant tolerance. Besides, the thymic output is seemingly not a beneficial biomarker for predicting cAMR; however, more in vivo and in vitro studies are needed for revealing the precise role of Tregs and RTEs in the transplantation context.

## 1. Introduction

During the advanced level of chronic kidney disease (CKD) which is called end-stage renal disease (ESRD), the patients usually need kidney replacement therapies, such as peritoneal dialysis, hemodialysis, or kidney transplantation. The majority of individuals who suffer from ESRD choose renal transplantation as an optimal treatment compared to dialysis. In recent decades, organ transplants have faced various obstacles, such as surgical restrictions and transplant rejection [1]. Some of these barriers have been resolved partially or entirely; for example, from the primary days of organ transplantation, immunosuppressive drugs have improved continually, which leads to a decrease in acute graft rejection by 12.2% [2]. However, chronic allograft rejection is still a serious obstacle against successful and long-term graft survival so that the 10-year survival of kidney transplant recipients falls below 45% and 55% in deceased and living donors, respectively [3]. Furthermore, despite the recent progressions, antibody-mediated rejection (AMR) is one of the main leading causes of graft rejection. In this circumstance, antibodies can target different molecules such as human leukocyte antigens (HLA), blood group antigens (ABO), and endothelial cells' antigens. Although the main problems in AMR are caused by antibodies, T cells also have crucial roles in the generation and maintenance of memory B cell responses. Nowadays, chronic antibody-mediated rejection (cAMR) is considered a significant cause of late allograft dysfunction in kidney transplantation [4].

Regulatory T (Treg) cells are the vital elements of the immune system which display a regulatory and suppressive function, and their activity leads to peripheral tolerance, limitation of inflammatory processes, and prevention of autoimmune diseases [5]. Due to the prominent role of Tregs in maintaining tolerance, transplant investigators have focused on the importance and application of Treg cells in organ transplantation. Several animal studies have demonstrated the importance of Tregs in the prevention of allograft rejection and the induction of graft tolerance. For example, it has been shown by Torrealba et al. that in the nonhuman primate model, recruitment of Treg cells to the transplanted kidney leads to metastable kidney transplant tolerance [6]. Also, Bozulic et al. have shown that Treg is an important player in the process of graft acceptance in long-term composite tissue allograft acceptors [7]. In clinical research, the role of these cells has been less understood and most of the shreds of evidence relied upon correlation studies. For example, Taflin et al. investigated the potential role of Tregs in control of the allogeneic response. They have found that the recruitment of Tregs during the acute phase of an allogeneic immune response can reduce the inflammatory processes and their subsequent graft damages [8]. Also, Bestard et al. revealed that the presence of Tregs in the biopsy of patients with subclinical renal allograft rejection could discriminate innocuous condition from ongoing rejection, and also, patients who had higher Treg in their allograft showed better renal function at both 2 and 3 years after transplantation [9]. Moreover, it has been shown that patients with subclinical rejection (SCR) without Treg have worse 5-year graft function in comparison to SCR patients who have Treg cells in their allograft and those patients without SCR [10]. Moreover, some researchers had found that follicular Treg (Tfr) proportion in both allograft and peripheral blood of cAMR patients was significantly lower than that of non-cAMR patients, and also, they figured out that consumption of sirolimus leads to the reduction of Tfr cell level, but the effect of cyclosporine A (CsA) and tacrolimus (Tac) on these cells was not statistically significant [11]. Totally, it seems that Treg cells have an essential role in allograft acceptance and long-term graft survival [12, 13].

Furthermore, some studies suggest a correlation between thymic output and transplant outcome. The thymus is one of the primary lymphoid organs known as the main place for maturation, selection of T cells, and production of normal T cells that are self-tolerant. After puberty, this organ gradually starts to involute and its connective tissue is progressively replaced by fatty tissue. The involution of the thymus causes the alteration of peripheral T cell subgroup distribution in such a way that the proportion of naive T cell pool diminishes and the share of memory T cells increases [14, 15]. Despite the involution and the reduction of thymus cells and tissues, this organ continues the production of a small number of T cells in adulthood, and this capacity varies between individuals. There are several ways for assessing the thymus activity, such as measuring the thymic mass by computed tomography (CT) scan, assessing thymic output by quantification of T cell receptor excision circles (TRECs) by real-time polymerase chain reaction (PCR), and measuring the frequency of recent thymic emigrants (RTEs) in peripheral blood by flow cytometry technique [16, 17]. RTEs are considered as the youngest subgroup of peripheral T cells, which has distinct function and phenotype characteristics from other groups of naïve T cell pool [18]. Various immunophenotyping markers have been proposed for the characterization of RTEs, but three clusters of differentiation (CD) markers that have been used frequently in previous studies are CD4^+^, CD31^+^, and CD45RA^+^ [19–21]. Several studies have revealed that specific circumstances lead to reactivation of the thymus, such as human immunodeficiency virus- (HIV-) infected patients who suffer from lymphodepletion after antiretroviral therapy and patients who receive intensive cytotoxic chemotherapy [22–25].

Recent studies have shown the potential role of the thymus in transplant tolerance [26–28]. Moreover, it has been shown that the thymic output of heart transplant patients can be considered as a critical element in the onset of AMR. Indeed, the proportion of RTEs in heart transplant patients with AMR has been significantly higher than that in the patient with cellular rejection or no evidence of rejection [17]. Another application and importance of the RTE in organ transplantation had been revealed by the study that showed the pretransplant measurement of RTE could have predicted the acute rejection in antithymocyte globulin- (ATG-) treated patients. In this study, patients with a higher percentage or absolute number of RTE showed a higher risk of acute rejection [19].

As most of the studies have investigated the role of Treg cells during cellular rejection and fewer pieces of evidence are available about the association of cAMR and Tregs, we decided to design this study to investigate the association of Treg cells and RTEs in kidney transplant recipients with cAMR. Also, to the best of our knowledge, no study has inquired about the association of RTE and cAMR in kidney transplanted patients.

## 2. Methods and Patients

### 2.1. Study Design

In this study, two groups were included, the stable graft function subjects (SGF, *n* = 20) and chronic antibody-mediated rejection patients (cAMR, *n* = 28). Both groups received immunosuppressive regimens including tacrolimus or cyclosporine, CellCept, and prednisolone. The SGF patients had no clinical or laboratory symptoms of graft rejection and cAMR patients had high creatinine concentration and low estimated glomerular filtration rate (eGFR), and patients in both groups had no previous history of infection at least six months before the sampling. All cAMR rejections were biopsy-proven. Intravenous immunoglobulin (IVIg) and rituximab were not used by patients in both groups. The kidney transplanted patients were recruited from the kidney transplantation unit of three university hospitals in Tehran. All participants fulfilled informed consent forms before sampling.

### 2.2. Cell Isolation

Peripheral blood was collected in tubes containing ethylenediaminetetraacetic acid (EDTA), and peripheral blood mononuclear cells (PBMCs) were isolated by Ficoll Lymphodex (Inno-Train, Germany) based on density gradient centrifugation and subsequently stored at a liquid nitrogen tank (-196°C) until performing flow cytometry tests.

### 2.3. Multicolor Flow Cytometry

**Figure 1:**
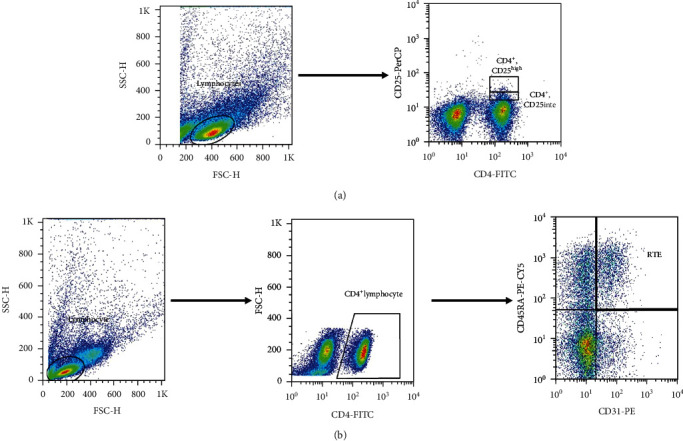
Gating strategy for Treg cells and RTE subpopulations. Gating strategy for detection of CD4^+^CD25^high^ Treg (a). Gating strategy for detection of CD4^+^, CD31^+^, CD45RA^+^ RTEs (b).

The thawing and refreshment procedure of PBMCs was performed by Roswell Park Memorial Institute (RPMI) 1640 medium (Biosera, USA) containing 10% FBS (Thermo Fisher Scientific, Gibco, USA); then, PBMCs were washed by washing buffer and eventually resuspended in phosphate-buffered saline (PBS). Suspended cells were stained by fluorochrome-conjugated antibodies based on their CD markers. Cells were stained by CD4-FITC, CD31-PE, and CD45RA-PE-CY5 from BioLegend (San Diego, CA) for quantifying the RTE population, and also, CD4-FITC and CD25-PerCP from BioLegend (San Diego, CA) were used to quantify the Treg population (Figure 1). The absolute number of each cell subset was calculated according to the dual-platform method, which is described by the World Health Organization (WHO) guideline, namely, by using complete blood count parameters obtained at the sampling, and the fraction of each cell subset was determined by flow cytometry [29].

### 2.4. Statistical Analysis

Statistical analyses have been done by Stata 13.0 software (StataCorp 2013; Stata Statistical Software: Release 13; College Station, TX: StataCorp LP), and Prism 6.0.1 (GraphPad Software, La Jolla California USA; http://www.graphpad.com) was used for graphical presentation. The Mann–Whitney *U* test was used for the between-group difference analysis, and the *P* value less than 0.05 (*P* < .05) was considered as statistically significant. Spearman's rank correlation test was used for correlation assessment.

## 3. Results

### 3.1. Basic Characteristics of the Study Groups

**Figure 2:**
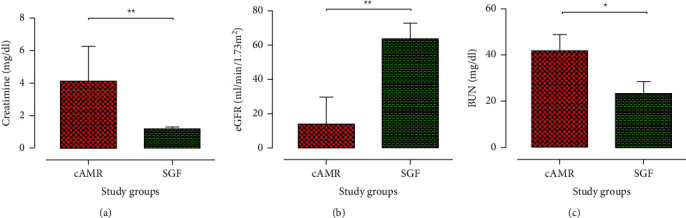
Renal function parameters in the study patients. The median of (a) serum creatinine concentration, (b) eGFR, and (c) BUN was used for kidney function assessment. cAMR patients displayed impaired kidney function, determined by higher serum creatinine and BUN levels and lower eGFR. Bar graphs show the median and interquartile range (IQR). Stable graft function (SGF) patients, *n* = 20; chronic antibody-mediated rejection (cAMR) group, *n* = 28. Intergroup differences were evaluated with the Mann–Whitney *U* test (^∗^*P* < 0.05; ^∗∗^*P* < 0.01).

Table 1 summarizes the demographic and clinical data of the included patients. SGF patients were under conventional immunosuppressive regimen (tacrolimus or cyclosporine/CellCept/prednisolone) and without any clinical and laboratory indication of graft rejection (mean creatinine level: 1.31 mg/dl, estimated glomerular filtration rate (eGFR): 63.18 ml/min/1.73 m^2^). Eighteen patients of the SGF group received allograft from deceased donors, and two received their allograft from living donors. The cAMR patients had high creatinine concentration (4.41 mg/dl) and low eGFR level (21.34 ml/min/1.73 m^2^) (Figures 2(a)–2(c)). Also, 23 patients in this group received their transplanted allograft from a deceased donor, and the rest of them received their allograft from living ones.

### 3.2. Distribution of T Cell Subsets in the Study Groups

**Figure 3:**
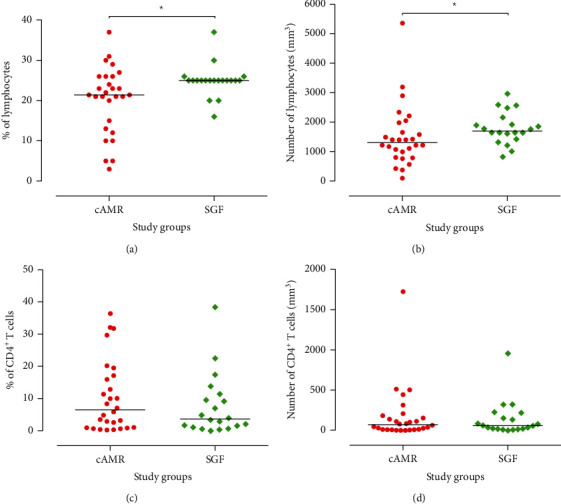
Lymphocyte and CD4^+^ T cell quantitative analysis in the study groups. Flow cytometric analysis of peripheral blood mononuclear cells (PBMCs) from stable graft function (SGF, *n* = 20) and chronic antibody-mediated rejection (cAMR, *n* = 28) patients. The percentage and number of (a, b) lymphocytes and (c, d) CD4^+^ T cells are displayed as individual values in each group. Intergroup differences were assessed by the Mann–Whitney *U* test. Horizontal lines show the median value (^∗^*P* < 0.05).

**Figure 4:**
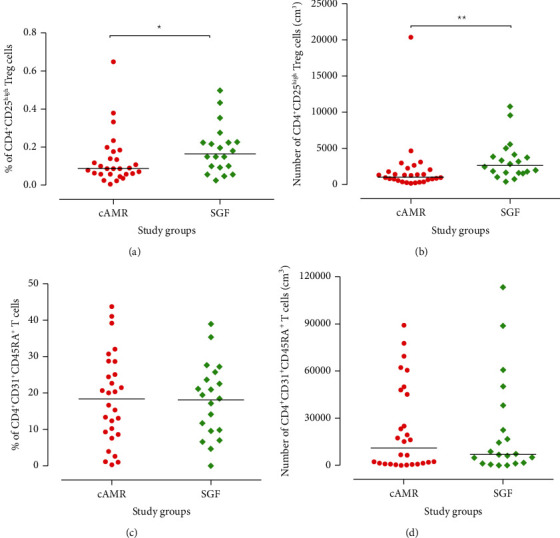
Distribution of Treg and RTE subsets among the study groups. Percentage and absolute number/cm^3^ of (a, b) Treg (CD4^+^CD25^high^) cells and recent thymic emigrant (RTE) cells are displayed as individual values in each study group. The absolute number of each subset was calculated based on the cell blood count at the sampling time and flow cytometric results according to the dual-platform method (described by WHO guideline). Intergroup differences were evaluated by the Mann–Whitney *U* test. Horizontal lines show the median value of each cell subset (^∗^*P* < 0.05; ^∗∗^*P* < 0.01).

The cAMR patients showed a remarkable decrease in the percentage and the absolute number of total lymphocytes (*P* = 0.03, Figures 3(a) and 3(b)). But the absolute number and percentage of circulating CD4^+^ T cells in SGF subjects were similar to those in cAMR patients (*P* = 0.95; *P* = 0.47, respectively)(Figures 3(c) and 3(d)). We used mice anti-human CD4 and CD25 antibodies as described in Methods and Patients, for analysis of the Treg cells. The percentage of CD4^+^CD25^high^ Treg cells was significantly lower in the cAMR patients (0.13%) than in SGF subjects (0.18%) (*P* = 0.05; Figure 4(a)). Also, the absolute number of CD4^+^CD25^high^ Tregs was significantly lower in cAMR patients in comparison to SGF subjects (2005.42 vs. 3369.03 cells per cm^3^ of blood) (*P* = 0.001; Figure 4(b)). Besides, we assessed the RTEs by mice anti-human CD4, CD31, and CD45RA antibodies and there was no significant difference in the percentage as well as the absolute number of this cell subset between cAMR and SGF patients (*P* = 0.96 and *P* = 0.92, respectively) (Figures 4(c) and 4(d)).

### 3.3. Calcineurin Inhibitors Affect the Percentage and the Absolute Number of CD4^+^CD25^high^ Treg Cells

**Figure 5:**
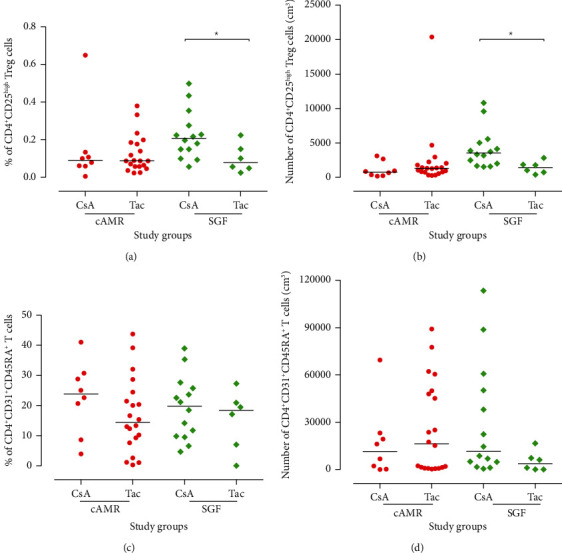
Subgrouping of CD4^+^CD25^high^ (Treg) and CD4^+^CD31^+^CD45RA^+^ (RTE) cell percentage based on the type of calcineurin inhibitor medication. Intergroup differences were evaluated with the Mann–Whitney *U* test (^∗^*P* < 0.05). Horizontal lines show the median value of cells. cAMR: chronic antibody-mediated rejection; SGF: stable graft function; Tac: tacrolimus; CsA: cyclosporine A.

We stratified the patients according to the types of calcineurin inhibitors (either Tac or CsA) in their immunosuppressive protocol. The percentage and absolute number/cm^3^ of CD4^+^CD25^high^ Treg and CD4^+^CD31^+^CD45RA^+^ RTE cells were compared between SGF subjects who received CsA with those who received Tac and also cAMR patients who received any type of these medications. The results showed that SGF subjects that received CsA had a higher percentage and number of CD4^+^CD25^high^ Treg cells than those who received Tac (*P* = 0.04 and *P* = 0.01, respectively), whereas the comparison of both Tac and CsA receivers in the cAMR group did not show any significant difference in the CD4^+^CD25^high^ Treg cells (Figures 5(a) and 5(b)). Moreover, both SGF and cAMR groups in none of the calcineurin inhibitor subsets did not show any significant difference in the number and percentage of RTEs (Figures 5(c) and 5(d)).

### 3.4. Correlation Analysis of Number of CD4^+^CD25^high^ Treg Cells and Lymphocytes

**Figure 6:**
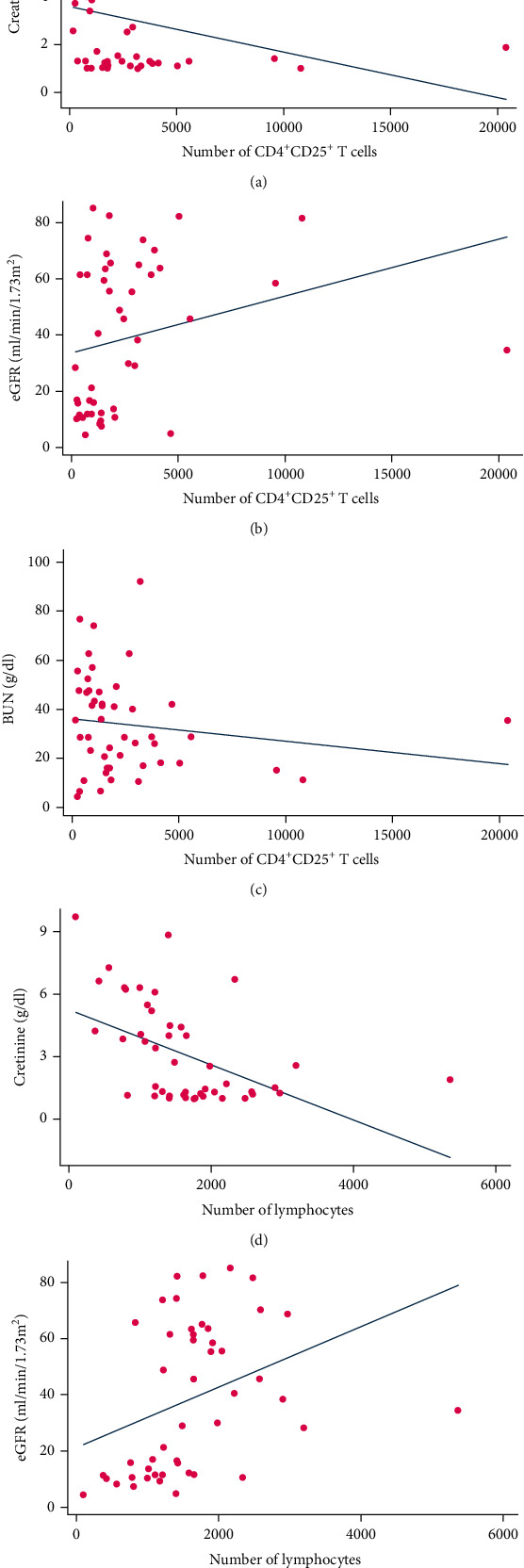
Correlation between the number of CD4^+^CD25^high^ Treg cells and lymphocytes and renal function parameters.

A significant low negative correlation (rho = −0.45, *P* = 0.002) and a significant low positive correlation (rho = 0.44, *P* = 0.002) were detected between the number of CD4^+^CD25^high^ Treg cells with creatinine and eGFR, respectively. Moreover, there is a significant moderate negative (rho = −0.52, *P* < 0.001) and a significant moderate positive correlation (rho = 0.53, *P* < 0.001) between the number of lymphocytes and creatinine concentration and eGFR level, respectively (Table 2, Figure 6).

## 4. Discussion

In organ transplantation, allograft rejection is considered the main obstacle against successful and long-term allograft survival. Allogeneic immune responses are the major causes of graft failure. Different immune cells such as B, T, neutrophil, NK, and dendritic cells are involved in the alloimmune responses. Nevertheless, the balance between effector and regulatory immune mechanisms can determine the fate of the transplanted organ. Treg cells are one of the most important regulatory cells which contribute to the control of different immune responses and graft tolerance [30]. Several studies have shown that Treg cells have an efficient role in long-term graft survival and assessment of peripheral and tissue infiltrated Tregs can be considered as a biomarker of tolerance [10, 31]. Several investigations have revealed that the presence and the frequency of intragraft Treg cells are an important factor for the prediction of transplant outcome in addition to immunosuppressive medications [9, 10, 32, 33]. Most of the research has focused on the association of these cells with both acute and chronic cellular rejection, but fewer data are available about the assessment of Tregs in AMR patients, especially cAMR ones. In this study, we aimed to assess the association of Tregs and RTEs with cAMR. Our data showed that the absolute number and percentage of Tregs were lower in the cAMR patients in comparison to SGF subjects (Figures 4(a) and 4(b)). Also, correlation analysis showed that patients with a higher number of Treg cells and lymphocytes have better allograft function. The results display that patients with higher Treg cells have higher eGFR (Figure 6(b)) and lower serum creatinine (Figure 6(a)). These data are consistent with previous studies that suggest the positive role of Treg cells in the maintenance of kidney graft survival. Although we showed the positive role of Treg in transplant outcome, the evaluation of their activity such as cytokine production, direct interaction with other immune cells, and their plasticity is an important question that should be answered in future studies. We found in our latest study that the mRNA level of TGF-*β* in cAMR subjects is significantly higher than that in SGF ones, and the strength of the relationship between TGF-*β* mRNA expression levels and renal function was very strong (Cohen's *d* = 1.26). Besides, the TGF-*β* protein level had a moderate relationship with renal function despite its nonsignificant difference between cAMR and SGF patients. In the case of IL-10, there was no significant difference between cAMR and SGF patients, but the serum level of both cytokines in each group was significantly higher than healthy subjects [34].

Because of the importance of Tregs in kidney transplantation, it seems necessary to investigate the influence of immunosuppressive treatment on this group of cells. Certain immunosuppressive agents can change the distribution, survival, and function of Tregs [35–37]. There are conflicting results about the influence of immunosuppressive drugs on Tregs especially in the case of calcineurin inhibitors (CNIs) such as CsA and Tac [35, 38]. Some studies have displayed that CNIs have a negative impact on the suppressive function of Tregs in allograft recipients [35, 39]. On the other hand, other studies have revealed that Treg cells of patients who received CNI are functional [40–42]. The mechanism of action of CsA is based on suppression of calcineurin-dependent IL-2 production that is an essential cytokine for the proliferation of T cells [43]. Some clinical and in vivo investigations revealed that treatment by CsA can promote the expression of Treg genes like Foxp3 [44, 45]. Also, another study has shown that patients who received CsA had a higher level of Tregs in comparison to healthy control [41]. Moreover, it has displayed that CsA significantly increases the percentage of Tregs among CD4^+^ cells and it shows that CsA increases the cross-linking of CD44 and hyaluronan that can promote Treg cell survival [42]. But the data about the effect of CNIs on Tregs are controversial and some other studies suggested the more beneficial impact of Tac on Treg cells [40, 46]. Our results showed that stable graft function patients who received CsA have a significantly higher level of Tregs in comparison to Tac receivers (Figures 5(a) and 5(b)). But there was no difference between CsA and Tac receivers in cAMR patients (Figures 5(a) and 5(b)). As mentioned previously, several studies have shown that CsA can increase the frequency and the expression of Treg genes like Foxp3 [41, 42, 44, 45], and our results in the SGF group were in line with these findings, although both Tac and CsA had a similar effect on the cAMR group. The difference between the effect of CsA on Tregs in cAMR patients compared to SGF individuals raises this doubt that Treg cells of cAMR patients may have an intrinsic defect and CsA cannot affect their functions and distribution and this issue suggests that isolation and in vitro study of the different function of Treg cells can improve our knowledge about the roles of these cells in the transplant rejection processes. Also, the level of total lymphocyte in peripheral blood of cAMR patients is less than that of SGF subjects (Figure 3(a)), which probably can be attributed to the infiltration of cells to the transplanted allograft.

Furthermore, recently, some studies have focused on the thymus function for predicting transplant outcome. RTE cells are the youngest subgroup of peripheral T cells, which are used for the assessment of thymic output [47]. Some studies suggest that thymic output can be a promising tool for predicting transplant rejection. To the best of our knowledge, this study is the first one that evaluated the level of RTEs in kidney transplant patients who endure cAMR. Our result did not show any significant difference between the RTE level of cAMR patients and SGF individuals (Figures 4(c) and 4(d)). Also, no significant difference was seen between the RTE level of CsA receivers and the patients who have taken Tac, in both cAMR and SGF groups (Figures 5(c) and 5(d)). As mentioned previously, RTE assessment in heart transplant patients has been able to predict the AMR. This controversy between this study and ours may be attributed to the types of immunosuppressive agents that heart transplant patients received. Moreover, patients in those studies [17, 19] which have shown the predictive role of RTEs in allograft rejection had received ATG. As the ATG is the T cell-depleting agent, it can induce the thymus for production of more newly generated T cells and this event can affect the balance of RTEs, but because the patients in our study had not received ATG, this difference may explain why they have a different proportion of RTE and no significant changes have occurred during the process of cAMR.

In conclusion, it seems that Treg cells have crucial roles in transplant tolerance maintenance along with immunosuppressive drugs. Although the thymic output had no significant association with the cAMR, possibly due to the low sample size, more in vivo and in vitro studies are needed with a larger sample size for understanding the precise role of RTEs in cAMR kidney transplant patients.

## Figures and Tables

**Table 1:** Demographic and clinical characteristics of the patients.

Variables	cAMR group (*n* = 28)	SGF group (*n* = 20)	*P* value
Age (years)	41.32 (14.62)	39.15 (10.17)	0.57
	40 [30.00, 51.50]	39 [34.00, 41.00]	
Sex (women)	9 (32.14)	6 (30.00)	0.88
Post-TX time	59.71 (64.39)	62.80 (35.92)	0.37
	39.00 [2.25, 84.00]	60.00 [36.00, 84.00]	
Weight (kg)	71.42 (17.24)	73.60 (9.79)	0.62
	72.50 [57.75, 83.25]	74.50 [73.00, 81.50]	
FBS (mg/dl)	121.35 (81.24)	115.45 (50.67)	0.81
	97.00 [86.50, 114.50]	100.50 [94.00, 116.00]	
Triglycerides (mg/dl)	120.00 (56.72)	131.70 (73.11)	0.53
	110.50 [90.00,145.00]	131.50 [95.00, 145.50]	
Cholesterol (mg/dl)	151.17 (30.12)	173.05 (51.29)	0.07
	150.00 [131.50, 165.50]	173.00 [151.50, 186.50]	
HDL (mg/dl)	47.54 (16.94)	47.00 (17.25)	0.96
	47.00 [36.00, 57.50]	47.00 [41.00, 55.50]	
LDL (mg/dl)	82.96 (26.16)	87.10 (40.59)	0.66
	84.00 [70.50, 102.00]	87.00 [62.00, 98.00]	
Sodium (Meq/I)	136.89 (6.03)	140.63 (3.16)	0.02
	136.50 [134.50, 140.50]	141.00 [139.00, 142.00]	
Potassium (Meq/I)	4.40 (0.84)	4.32 (1.18)	0.79
	4.35 [4.00, 4.90]	4.19 [3.80, 4.30]	
Phosphorus (mg/dl)	5.11 (1.69)	3.69 (0.90)	0.001
	4.90 [3.90, 5.80]	3.70 [3.15, 3.84]	
Calcium (mg/dl)	8.40 (0.87)	9.27 (0.31)	<0.001
	8.55 [7.75, 9.00]	9.30 [9.15, 9.45]	
Uric acid (mg/dl)	7.69 (2.61)	5.58 (1.51)	0.002
	7.35 [6.15, 9.15]	5.50 [4.95, 5.85]	
Urea (mg/dl)	83.75 (42.39)	41.16 (16.93)	<0.001
	89.50 [53.00, 108.50]	39.00 [30.00, 48.00]	
BUN (mg/dl)	37.68 (1.88)	25.70 (14.41)	0.02
	41.80 [23.65, 48.48]	23.28 [25.96, 28.60]	
eGFR (ml/min/1.73 m^2^)	21.35 (17.06)	63.17 (16.13)	<0.001
	13.81 [10.34, 29.36]	63.53 [58.85, 71.97]	
Creatinine (mg/dl)	4.41 (2.31)	1.31 (0.66)	<0.001
	4.10 [2.55, 6.26]	1.18 [1.07, 1.30]	
WBC (∗10^3^/*μ*l)	7.44 (3.41)	6.66 (1.20)	0.33
	6.30 [5.20, 9.20]	6.60 [5.70, 7.50]	
Platelet (∗10^3^/*μ*l)	185.03 (86.59)	218.20 (60.23)	0.15
	180.50 [125.00, 229.50]	218.00 [206.00, 263.00]	
Lymphocyte (%)	20.24 (8.34)	25.00 (3.99)	0.02
	21.41 [14.00, 26.00]	25.00 [25.00, 25.00]	
Hemoglobin (g/dl)	9.98 (2.14)	13.48 (2.97)	<0.001
	9.75 [8.15, 11.47]	13.85 [13.50, 15.30]	
Hematocrit (%)	32.02 (7.23)	40.27 (7.42)	<0.001
	30.50 [27.20, 34.75]	40.20 [38.55, 46.25]	

Scale and nominal variables were depicted as mean (SD) and median [Q1; Q3] and number (percent), respectively. The Mann–Whitney *U* test was used for the evaluation of intergroup differences. Abbreviations: cAMR: chronic antibody-mediated rejection; SGF: stable graft function; FBS: fasting blood sugar; HDL: high-density lipoprotein; LDL: low-density lipoprotein; BUN: blood urine nitrogen; eGFR: estimated glomerular filtration rate; WBC: white blood cell.

**Table 2:** Correlation of the number of CD4^+^CD25^high^ Treg cells and lymphocytes with renal function parameters.

Parameter	Number of CD4^+^CD25^high^ Treg cells	Interpretation	Number of lymphocytes	Interpretation
Creatinine	*ρ* = −0.45 *P* = 0.002	Low negative correlation	*ρ* = −0.52 *P* < 0.001	Moderate negative correlation
eGFR	*ρ* = −0.44 *P* = 0.002	Low positive correlation	*ρ* = −0.53 *P* < 0.001	Moderate positive correlation
BUN	*ρ* = −0.28 *P* = 0.05	Trivial	*ρ* = −0.002 *P* = 0.99	Trivial

eGFR: estimated glomerular filtration rate; BUN: blood urea nitrogen; *ρ*: Spearman's rank correlation coefficient or Spearman's rho.

## Data Availability

Data are available on request.

## References

[1] Fleming G. M. (2014). Renal replacement therapy review: past, present and future. *Organogenesis*.

[2] Hart A., Smith J. M., Skeans M. A. (2019). OPTN/SRTR 2017 annual data report: kidney. *American journal of transplantation*.

[3] US Renal Data System 2013 Annual Report End-stage renal disease in the United States. https://www.usrds.org/atlas13.aspx.

[4] Sun Q., Yang Y. (2013). Late and chronic antibody-mediated rejection: main barrier to long term graft survival. *Clinical and Developmental Immunology*.

[5] Vignali D. A. A., Collison L. W., Workman C. J. (2008). How regulatory T cells work. *Nature Reviews Immunology*.

[6] Torrealba J. R., Katayama M., Fechner J. H. (2004). Metastable tolerance to rhesus monkey renal transplants is correlated with allograft TGF-*β*1+CD4+T regulatory cell infiltrates. *The Journal of Immunology*.

[7] Bozulic L. D., Wen Y., Xu H., Ildstad S. T. (2011). Evidence that FoxP3+ regulatory T cells may play a role in promoting long-term acceptance of composite tissue allotransplants. *Transplantation*.

[8] Taflin C., Nochy D., Hill G. (2010). Regulatory T cells in kidney allograft infiltrates correlate with initial inflammation and graft function. *Transplantation*.

[9] Bestard O., Cruzado J. M., Rama I. (2008). Presence of FoxP3+ regulatory T cells predicts outcome of subclinical rejection of renal allografts. *Journal of the American Society of Nephrology*.

[10] Bestard O., Cuñetti L., Cruzado J. M. (2011). Intragraft regulatory T cells in protocol biopsies retain foxp3 demethylation and are protective biomarkers for kidney graft outcome. *American journal of transplantation*.

[11] Chen W., Bai J., Huang H. (2017). Low proportion of follicular regulatory T cell in renal transplant patients with chronic antibody-mediated rejection. *Scientific Reports*.

[12] Gorzin F., Amirzargar A. A., Mahmoudi M. J. (2017). FOXP3, ROR*γ*t and IL-10 cytokine profile in chronic heart failure. *Bratislavske lekarske listy*.

[13] Nikoueinejad H., Amirzargar A., Sarrafnejad A. (2014). Dynamic changes of regulatory T cell and dendritic cell subsets in stable kidney transplant patients: a prospective analysis. *Iranian journal of kidney diseases*.

[14] Crepin T., Carron C., Roubiou C. (2015). ATG-induced accelerated immune senescence: clinical implications in renal transplant recipients. *American journal of transplantation*.

[15] Gruver A. L., Hudson L. L., Sempowski G. D. (2007). Immunosenescence of ageing. *The Journal of pathology*.

[16] Dion M. L., Sekaly R. P., Cheynier R. (2007). Estimating thymic function through quantification of T-cell receptor excision circles. *Methods in molecular biology*.

[17] Sannier A., Stroumza N., Caligiuri G. (2018). Thymic function is a major determinant of onset of antibody-mediated rejection in heart transplantation. *American journal of transplantation*.

[18] Berkley A. M., Hendricks D. W., Simmons K. B., Fink P. J. (2013). Recent thymic emigrants and mature naive T cells exhibit differential DNA methylation at key cytokine loci. *Journal of Immunology*.

[19] Bamoulid J., Courivaud C., Crepin T. (2016). Pretransplant thymic function predicts acute rejection in antithymocyte globulin-treated renal transplant recipients. *Kidney international*.

[20] Batorov E. V., Tikhonova M. A., Kryuchkova I. V. (2017). CD4(+) memory T cells retain surface expression of CD31 independently of thymic function in patients with lymphoproliferative disorders following autologous hematopoietic stem-cell transplantation. *International journal of hematology*.

[21] Greinix H. T., Kuzmina Z., Weigl R. (2015). CD19+CD21low B cells and CD4+CD45RA+CD31+ T cells correlate with first diagnosis of chronic graft-versus-host disease. *Biology of Blood and Marrow Transplantation*.

[22] Nikolich-Zugich J. (2008). Ageing and life-long maintenance of T-cell subsets in the face of latent persistent infections. *Nature reviews Immunology*.

[23] Chaudhry M. S., Velardi E., Dudakov J. A., van den Brink M. R. (2016). Thymus: the next (re)generation. *Immunological Reviews*.

[24] Ruiz-Mateos E., Rubio A., Vallejo A. (2004). Thymic volume is associated independently with the magnitude of short- and long-term repopulation of CD4+ T cells in HIV-infected adults after highly active antiretroviral therapy (HAART). *Clinical and Experimental Immunology*.

[25] McFarland R. D., Douek D. C., Koup R. A., Picker L. J. (2000). Identification of a human recent thymic emigrant phenotype. *Proceedings of the National Academy of Sciences of the United States of America*.

[26] Yamada K., Gianello P. R., Ierino F. L. (1997). Role of the thymus in transplantation tolerance in miniature swine. I. Requirement of the thymus for rapid and stable induction of tolerance to class I-mismatched renal allografts. *The Journal of experimental medicine.*.

[27] Yamada K., Shimizu A., Utsugi R. (2000). Thymic transplantation in miniature swine. II. Induction of tolerance by transplantation of composite thymokidneys to thymectomized recipients. *Journal of Immunology*.

[28] Kamano C., Vagefi P. A., Kumagai N. (2004). Vascularized thymic lobe transplantation in miniature swine: thymopoiesis and tolerance induction across fully MHC-mismatched barriers. *Proceedings of the National Academy of Sciences of the United States of America*.

[29] World Health Organization ROfS-EA (2009). *Laboratory guidelines for enumeration CD4 T lymphocytes in the context of HIV/AIDS (revised version 2009)*.

[30] Kondelkova K., Vokurkova D., Krejsek J., Borska L., Fiala Z., Ctirad A. (2010). Regulatory T cells (TREG) and their roles in immune system with respect to immunopathological disorders. *Acta Medica (Hradec Králové)*.

[31] Mirzakhani M., Shahbazi M., Akbari R. (2020). Reduced CD4^+^ CD25^++^ CD45RA^−^ Foxp3^hi^ activated regulatory T cells and its association with acute rejection in patients with kidney transplantation. *Transplant Immunology.*.

[32] Grimbert P., Mansour H., Desvaux D. (2007). The regulatory/cytotoxic graft-infiltrating T cells differentiate renal allograft borderline change from acute rejection. *Transplantation*.

[33] Bestard O., Cruzado J. M., Mestre M. (2007). Achieving donor-specific hyporesponsiveness is associated with FOXP3+ regulatory T cell recruitment in human renal allograft infiltrates. *Journal of Immunology*.

[34] Salehi S., Shahi A., Afzali S. (2020). Transitional immature regulatory B cells and regulatory cytokines can discriminate chronic antibody-mediated rejection from stable graft function. *International Immunopharmacology*.

[35] De Serres S. A., Sayegh M. H., Najafian N. (2009). Immunosuppressive drugs and Tregs: a critical evaluation!. *Clinical Journal of the American Society of Nephrology*.

[36] Jamali S., Sarafnejad A., Ahmadpoor P. (2019). Sirolimus vs mycophenolate moftile in Tacrolimus based therapy following induction with antithymocyte globulin promotes regulatory T cell expansion and inhibits ROR*γ*t and T-bet expression in kidney transplantation. *Human immunology*.

[37] Karimi M., Ahmadpoor P., Nafar M. (2020). Frequency of dendritic cell subsets and ILT3, ILT4 gene expression in two different immunosuppressive protocols in kidney transplant recipients. A cohort report. *Molecular Biology Reports*.

[38] Fanigliulo D., Lazzerini P. E., Capecchi P. L., Ulivieri C., Baldari C. T., Laghi-Pasini F. (2015). Clinically-relevant cyclosporin and rapamycin concentrations enhance regulatory T cell function to a similar extent but with different mechanisms: an in-vitro study in healthy humans. *International Immunopharmacology*.

[39] Akimova T., Kamath B. M., Goebel J. W. (2012). Differing effects of rapamycin or calcineurin inhibitor on T-regulatory cells in pediatric liver and kidney transplant recipients. *American journal of transplantation*.

[40] Miroux C., Morales O., Ghazal K. (2012). In vitro effects of cyclosporine A and tacrolimus on regulatory T-cell proliferation and function. *Transplantation*.

[41] Brandt C., Pavlovic V., Radbruch A., Worm M., Baumgrass R. (2009). Low-dose cyclosporine A therapy increases the regulatory T cell population in patients with atopic dermatitis. *Allergy*.

[42] Ruppert S. M., Falk B. A., Long S. A., Bollyky P. L. (2015). Regulatory T cells resist cyclosporine-induced cell death via CD44-mediated signaling pathways. *International Journal of Cell Biology*.

[43] Russell G., Graveley R., Seid J., Al-Humidan A.-K., Skjodt H. (1992). Mechanisms of action of cyclosporine and effects on connective tissues. *Seminars in arthritis and rheumatism*.

[44] Knol E. F., Haeck I. M., van Kraats A. A. (2012). Modulation of Lymphocyte Function _In Vivo_ via Inhibition of Calcineurin or Purine Synthesis in Patients with Atopic Dermatitis. *The Journal of investigative dermatology*.

[45] Li Z. Y., Wu Q., Yan Z. (2013). Prevention of acute GVHD in mice by treatment with Tripterygium hypoglaucum Hutch combined with cyclosporin A. *Hematology*.

[46] Miroux C., Moralès O., Carpentier A. (2009). Inhibitory effects of cyclosporine on human regulatory T cells in vitro. *Transplantation proceedings*.

[47] Houston E. G., Higdon L. E., Fink P. J. (2011). Recent thymic emigrants are preferentially incorporated only into the depleted T-cell pool. *Proceedings of the National Academy of Sciences of the United States of America*.

